# FSL-1, a bacterial-derived toll-like receptor 2/6 agonist, enhances resistance to experimental HSV-2 infection

**DOI:** 10.1186/1743-422X-6-195

**Published:** 2009-11-10

**Authors:** William A Rose, Chris L McGowin, Richard B Pyles

**Affiliations:** 1 Department of Microbiology and Immunology, University of Texas Medical Branch, Galveston, TX, USA; 2 Department of Infectious Diseases, LSU Health Science Center, New Orleans, LA, USA; 3 Department of Pediatrics, University of Texas Medical Branch, Galveston, TX, USA

## Abstract

**Background:**

Herpes simplex virus type 2 (HSV-2) is a leading cause of genital ulceration that can predispose individuals to an increased risk of acquiring other sexually transmitted infections. There are no approved HSV-2 vaccines and current suppressive therapies require daily compound administration that does not prevent all recurrences. A promising experimental strategy is the use of toll-like receptor (TLR) agonists to induce an innate immune response that provides resistance to HSV-2 infection. Previous studies showed that anti-herpetic activity varied based on origin of the agonists and activation of different TLR indicating that activity likely occurs through elaboration of a specific innate immune response. To test the hypothesis, we evaluated the ability of a bacterial-derived TLR2/6 agonist (FSL-1) to increase resistance to experimental genital HSV-2 infection.

**Methods:**

Vaginal application of FSL-1 at selected doses and times was evaluated to identify potential increased resistance to genital HSV-2 infection in the mouse model. The FSL-1 induced cytokine profile was quantified using kinetically collected vaginal lavages. Additionally, cytokine elaboration and organ weights were evaluated after single or multiple FSL-1 doses to establish a preliminary safety profile. Human vaginal EC cultures were used to confirm the mouse model outcomes.

**Results:**

The results showed that vaginally-applied FSL-1 created an environment resistant to a 25-fold higher HSV-2 challenge dose. Mechanistically, vaginal FSL-1 application led to transient elaboration of cytokines linked to anti-herpetic innate immune responses. No gross local or peripheral immunotoxicity was observed even after multiple dosing. FSL-1 also created an anti-herpetic environment in cultures of human vaginal epithelial cells (EC).

**Conclusion:**

The results showed, for the first time, that the bacterial-derived TLR2/6 agonist FSL-1 induced significant resistance to HSV-2 infection when applied in mice or human vaginal EC cultures. Cytokine evaluation illustrated that anti-herpetic activity correlated with induction of a specific profile. The identified anti-herpetic profile provides an invaluable resource for the future design of novel compounds to reduce genital HSV-2 transmission and improves understanding of the complex innate immune response to potential pathogens elicited by the vaginal mucosa.

## Introduction

Genital herpes simplex virus type 2 (HSV-2) is one of the most wide spread sexually transmitted infections (STI) and prevalence varies from 16-97% based on age, culture, ethnicity, geographic location, sex and other factors [1-3]. Following genital infection, HSV-2 establishes a life-long latency in sacral ganglia and periodically can reactivate resulting in the formation of genital lesions [1,4]. Controlling the spread of HSV-2 is important because the genital lesions can serve as portals of entry for other STI including human immunodeficiency virus type 1 (HIV-1) [1,5-7]. Also important to transmission cycles, HSV-2 is frequently shed from the genital tract in the absence of symptoms [8]. Recent studies showed that HSV-2 is associated with coinfections and can exacerbate the pathologies produced by STI [1,5-7]. So prevention of transmission is a laudable goal, however, there are no Food and Drug Administration-approved vaccines for HSV-2 [1,9]. Further, treatment of existing infections involves daily antiviral therapy that only suppresses but does not completely prevent all recurrences [1,10]. New approaches are needed that can prevent or reduce the likelihood of transmission.

One promising experimental strategy is the use of evolutionarily conserved pathogen structures termed pathogen-associated molecular patterns (PAMP) that are recognized by toll-like receptors (TLR) [11,12]. Each TLR alone or in a heterodimer binds to a specific PAMP and based on the ligand initiates a particular signaling transduction cascade [11,12]. TLR signaling elicits cytokine production and leukocyte recruitment resulting in a highly coordinated innate immune response against genital HSV-2 infection [11,13]. Previous work showed that viral-associated TLR agonists vaginally-applied prior to challenge increased resistance to HSV-2 infection in mice [14-19]. Polyinosinic-polycytidylic acid (PIC), a synthetic TLR3 agonist applied 24 h prior to viral inoculation significantly increased resistance to genital HSV-2 infection and mice that were infected showed significantly delayed disease signs and increased survival times [17]. Similar anti-herpetic activity was observed following application of imiquimod (IMI) or unmethylated CpG oligonucleotides (CpG), synthetic TLR7/8 or 9 agonists, respectively [14-16,18,19]. Interestingly, murine experiments using bacterial-derived TLR agonists including peptidoglycan (PGN; TLR2), lipopolysaccharide (LPS; TLR4) or flagellin (FLAG; TLR5) showed no significant induction of resistance to HSV-2 [14,15]. In a recent study, FimH derived from *Escherichia coli *elicited significant resistance to genital HSV-2 infection through TLR4 activation and IFNβ production [20]. The varying activity observed following TLR agonist application indicates that establishment of an HSV-2 resistant environment likely occurs through activation of a specific innate immune response.

To test the hypothesis that HSV-2 resistance could be engendered through elaboration of a specific cytokine response, we selected a synthetic diacyl lipopeptide derived from *Mycoplasma salivarium*, fibroblast stimulating ligand-1 (FSL-1), that is recognized by TLR2/6 heterodimers [21,22]. TLR2 and 6 are highly expressed by human [13] and murine [23] vaginal and lower genital tract epithelial cells (EC). Studies showed that HSV-2 is recognized through TLR2-mediated binding of the glycoprotein spikes within the viral lipid envelope [11,24]. Further, activation of TLR2/6 by FSL-1 can create an environment that is less susceptible to infection by other bacteria through elaboration of a specific cytokine profile [25]. Evaluation of the HSV-2-resistant vaginal environment in specific knockout mice or in animals treated with TLR agonists of viral origin has identified a cytokine profile that includes IL-2, IL-12(p40), IFNα, IFNβ, IFNγ and CCR5 chemokines [15-17,19]. Additionally, recent work with primary and immortalized human vaginal EC cultures showed that FSL-1 elicited innate immune responses similar to PIC [13], therefore we hypothesized that the FSL-1-elaborated cytokine pattern would engender resistance to HSV-2 infection in the mouse model and in human vaginal EC.

FSL-1 was tested at several doses and application times in the mouse model and in human vaginal EC cultures to evaluate potential induced resistance to genital HSV-2 infection. Cytokine elaboration induced by FSL-1 was quantified for comparison to other TLR agonists to identify an innate immune response profile associated with anti-herpetic activity. Kinetic cytokine elaboration elicited by a single or by multiple doses of FSL-1 was quantified and weights of relevant organs were measured to establish a preliminary safety profile of the induced innate immune response for the rational design of future anti-herpetic compounds. Additionally, the impact of FSL-1 on HSV-2 replication in human vaginal EC was established to confirm that potential anti-herpetic activity was not specific to the murine vaginal environment. Collectively, the results indicated that FSL-1 induced significant resistance to experimental genital HSV-2 infection through elaboration of a specific cytokine response profile.

## Materials and methods

### Cell culture and HSV-2 propagation

Immortalized human vaginal EC from three different donors (V11I, V12I, V19I) were cultured in a 1:1 mixture of VEC-100 (MatTek, Ashland, MA) and keratinocyte serum-free medium (Invitrogen, Carlsbad, CA) as described previously [13]. HSV-2 strain 186 [26], prepared from infected Vero cell cultures (ATCC CCL81), was used for all experiments. Viral stocks were titered as described previously [18] and stored at -80°C.

### Mouse model of genital HSV-2 infection and dose-ranging paradigm

Female Swiss-Webster mice (Harlan Sprague Dawley, Indianapolis, IN) weighing 20-25 g were housed in Association for Assessment and Accreditation of Laboratory Animal Care-approved quarters and provided with unlimited access to food and water. All procedures were performed humanely and approved by the University of Texas Medical Branch Institutional Animal Care and Use Committee. Following a 7d acclimation to the facility, mice were hormonally-conditioned by subcutaneous delivery of 150 mg/kg of Depo-Provera (UpJohn, Kalamazoo, MI) 7d prior to viral inoculation as described previously [17,18].

FSL-1 was diluted in Dulbecco's Phosphate Buffered Saline (DPBS) without calcium or magnesium (Cellgro, Herndon, VA) to achieve a 0.1 μg/μL solution and delivered vaginally using a positive displacement pipet (Rainin, Oakland, CA) at 2 or 6 μg/mouse prior to or following viral challenge as specified for each experiment. Dosing was based on FSL-1 solubility in the DPBS vehicle and the volumetric constraints of the murine vagina resulting in a maximum delivery of 6 μg. DPBS vehicle treated mice were followed in parallel to serve as controls for all experiments. Mice were swabbed with a wet then dry calcium alginate swab (Fisher, Pittsburgh, PA) just prior to instilling 25 μL of HSV-2 strain 186 (10^4^pfu/mouse) vaginally using a positive displacement pipet. The challenge dose, a 100 times greater than the dose required to cause lethal outcomes in 50% of the mice (LD_50_) [17,18], was selected to ensure that enough mice in the DPBS vehicle treatment group exhibited disease signs to allow for statistical comparisons.

FSL-1-induced resistance to HSV-2 infection was quantified using the infectious dose 50% (ID_50_) shift paradigm [17]. Briefly, FSL-1 doses of 2 or 6 μg/mouse were delivered at indicated times and then groups of mice were challenged with viral inoculums of 10^2^, 10^3 ^or 10^4^pfu (n = 10). DPBS treated groups were employed to control for the potential impact of the vehicle on HSV-2 infection and were challenged with viral inoculums of 10^1^, 10^2 ^or 10^3^pfu/mouse (n = 10). Infected animals were identified by plating of vaginal swabs collected 2d after viral challenge [27]. Mice were assessed daily (d1-14) for the appearance of disease signs and followed for survival as described previously [17,18].

### Cytokine quantification

Vaginal lavages were collected at 0 and 24 h after application of FSL-1 (6 μg) or an equivalent volume of the DPBS vehicle (60 μL) at 6 and 48 h after treatments from a second group of mice (n = 5 mice/treatment for each group). Lavages were collected as described previously [17] and collections were scheduled to prevent an artificial "washout" of vaginal cytokines by only sampling an animal once within a 24 h period. For repetitive dosing evaluations, vaginal lavages were collected from FSL-1 (6 μg) or DPBS vehicle (60 μL) treated mice at 6 h (n = 5 each; group 1) or 24 h (n = 5 each; group 2) after a once daily application for 5 consecutive days. Vaginal cytokines in the recovered lavages (~100 μL) were quantified using the BioPlex Mouse Group I cytokine kit (Bio-Rad, Hercules, CA) or by ELISA for mouse IFNα or IFNβ (PBL, New Brunswick, NJ). Absolute cytokine quantities (pg/mL) were extrapolated from a standard curve run in parallel.

### Quantification of FSL-1 induced HSV-2 resistance in cultured human vaginal epithelial cells

V11I, V12I or V19I immortalized human vaginal EC were seeded into 96 well culture plates at 1 × 10^5^cells/well in 100 μL of medium and incubated overnight. FSL-1 (6 μg or 0.1 μg) or the DPBS vehicle (10 μL) were added at 24, 6 or just prior to HSV-2 inoculation (10^4^pfu/well). At 24 h post-infection (PI) the contents of each well were collected (100 μL) and 50 μL was used for plaque titration assays to quantify viral titer [18]. DNA was extracted from the remaining 50 μL using the Qiagen DNeasy 96 Kit (Qiagen, Valencia, CA) and subjected to quantitative TaqMan PCR that targeted the HSV-2 glycoprotein B gene as described previously [28].

### Statistical analyses

Data were analyzed by specific statistical tests as indicated in the results section using the Prism software package v4.0 (Graph Pad, San Diego, CA).

## Results

### FSL-1 impacted genital HSV-2 infections in mice

FSL-1 doses were applied vaginally at selected time points relative to viral challenge to identify doses and delivery times that could induce significant resistance to HSV-2 infection. Initially, a single application of FSL-1 (2 μg) delivered at 24 or 6 h (n = 10 mice/group) prior to HSV-2 inoculation (10^4^pfu) was evaluated based on efficacy results from experimentation with other TLR agonists [14,17]. FSL-1, delivered at either time, significantly (p < 0.05; Student's t-test) delayed the appearance of disease signs (disease incidence) and increased survival times compared to DPBS vehicle treated controls (Table 1).

**Table 1:** Vaginal application of FSL-1 significantly delayed HSV-2 disease development and increased survival times.

FSL-1 and vehicle groups^a^	Time to symptoms^b^	Survival time^c^	Survival^d^
	(days)	(days)	(%)
FSL-1 2 μg 24 h prior	8.0 (6.4-9.7)^e, f^	9.4 (7.8-11.0)^e, f^	1/10 (10)
FSL-1 2 μg 6 h prior	9.0 (8.1-9.9)^e, f^	10.1 (8.9-11.3)^e, f^	0/10 (0)
FSL-1 2 μg 1 h prior, 1 h after	6.0 (5.5-6.4)	7.3 (6.8-7.7)	0/10 (0)
FSL-1 2 μg 6 h, 5 h, 4 h prior	6.4 (5.9-7.0)	9.1 (7.0-11.2)	1/9 (11)
FSL-1 6 μg 6 h prior	8.5 (6.7-9.9)^e, f^	10.0 (8.4-11.6)^e, f^	4/10 (40)
DPBS vehicle control	5.8 (5.0-6.4)	7.2 (6.9-7.6)	0/8 (0)

^a ^Intravaginal application of FSL-1 or DPBS vehicle control at selected times prior to or after vaginal inoculation of HSV-2 (10^4^pfu).

^b ^Mean (95% confidence interval) for day that each mice first showed disease signs within 14d PI

^c ^Mean (95% confidence interval) for day that each mice succumbed to disease within 14d PI.

^d ^Number of mice that did not succumb to disease within 14d PI/total number of animals in the group (percent survival).

^e ^p < 0.05 compared to DPBS vehicle control (ANOVA, Dunnett's Test).

^f ^p < 0.05 compared to FSL-1 2 μg 1 h prior, 1 h after (ANOVA, Dunnett's Test).

An additional dosing regimen of FSL-1 was evaluated by employing a potential microbicide delivery schedule consisting of application prior to or just after a sexual encounter [29]. FSL-1 (2 μg/dose) was applied vaginally 1 h prior and 1 h after HSV-2 inoculation (10^4^pfu) to a separate group of mice (n = 10). Interestingly, this treatment did not impact HSV-2 disease incidence nor alter survival times compared to DPBS vehicle treatment and was significantly (p < 0.05; Student's t-test) less effective than FSL-1 (2 μg) applied at 24 or 6 h prior to viral challenge (Table 2). In an additional study, 2 other groups of mice received 3 repetitive FSL-1 doses (2 μg/dose) at 6, 5 and 4 h prior to HSV-2 inoculation or a single 6 μg dose 6 h prior to viral challenge. The single FSL-1 (6 μg) dose produced significantly (p < 0.05; Student's t-test) improved outcomes compared to DPBS vehicle application or FSL-1 (2 μg) applied at 1 h prior and 1 h after viral inoculation (Table 1) indicating a time dependence for FSL-1-mediated resistance to HSV-2.

**Table 2:** FSL-1 application significantly protected against genital HSV-2 challenge in mice.

HSV-2 dose	**FSL-1 2 μg 24 h prior**^a^		FSL-1 2 μg 6 h prior		DPBS vehicle control	
	
(pfu)	**%Infected**^b^	**%Survival**^c^	%Infected	%Survival	%Infected	%Survival
1 × 10^1^	ND^d^	ND	ND	ND	30	70
1 × 10^2^	30	70	10^e^	90	70	50
1 × 10^3^	50^e^	50^e^	100	40	100	0
1 × 10^4^	90	10	100	0	ND	ND

**ID_50_^f^**	760 (80-7000)		260 (10-6000)		31 (10-80)	
**LD_50_^g^**	760 (80-7000)		660 (300-1000)		68 (7-700)	

^a ^Intravaginal application of FSL-1 or DPBS vehicle control at indicated times prior to vaginal HSV-2 inoculation (n = 10 mice/treatment at each HSV-2 dose).

^b ^Percentage of mice with infectious virus in d2 vaginal swabs.

^c ^Percentage of mice that survived HSV-2 vaginal challenge up to d14 PI.

^d ^ND, not determined.

^e ^p < 0.05 compared to vehicle control (Fisher's exact test).

^f ^Dose of the virus required to infect 50% of the mice based on %Infected data; ID_50 _(95% confidence interval).

^g ^Dose of the virus required to cause lethal outcomes in 50% of the mice based on % survival data; LD_50 _(95% confidence interval).

### Vaginal FSL-1 application increased resistance to HSV-2 by 25-fold

Having identified 2 effective treatment times (24 and 6 h prior to inoculation), the ID_50 _shift paradigm [17] was employed to quantify the resistance to HSV-2 challenge afforded by FSL-1. Single doses of 2 or 6 μg per mouse were evaluated at the 2 time points to identify the most effective FSL-1 regimen (n = 30 mice per treatment). Treated animals then were distributed randomly into subgroups (n = 10) for subsequent challenge with escalating viral doses (10^2^, 10^3^, or 10^4^pfu) (Table 2). FSL-1 (2 or 6 μg) delivered at 24 or 6 h prior to viral inoculation resulted in similar outcomes as observed in Table 1 when genitally challenged with 10^4^pfu of HSV-2 confirming the previous results. Of the tested doses and times, the lower dose of FSL-1 (2 μg) applied 24 h prior to HSV-2 inoculation produced a significant shift in the ID_50 _(760 pfu) resulting in a 25-fold increase in resistance to viral infection compared to DPBS vehicle treated control animals (ID_50 _= 31 pfu). For the animals that were infected, the treatment also resulted in a 10-fold increase in survival (LD_50 _= 760 pfu). FSL-1 (2 μg) delivered 6 h prior to HSV-2 challenge increased the ID_50 _(260 pfu) and LD_50 _(660 pfu) by 10-fold compared to DPBS vehicle control (Table 2). Application of FSL-1 (6 μg) at 24 or 6 h prior to viral inoculation produced similar increases in the ID_50 _and LD_50 _compared to FSL-1 (2 μg) delivered 6 h prior to HSV-2 challenge. We examined the cytokine profile elaborated by FSL-1 application to better understand how a bacterial-derived TLR agonist modulated the innate immune response to provide resistance to genital HSV-2 challenge.

### FSL-1-elicited cytokines were consistent with innate responses previously associated with resistance to genital HSV-2 infection

**Figure 1:**
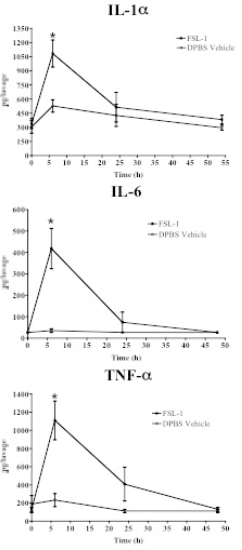
**FSL-1 transiently induced pro-inflammatory cytokines**. Selected cytokines were quantified kinetically from murine vaginal lavages (n = 5/treatment) collected 0, 6, 24 and 48 h after FSL-1 (6 μg; closed square) or DPBS vehicle (60 μL; open circle) application. The pro-inflammatory cytokines IL-1α, IL-6 and TNF-α were significantly increased (*, p < 0.05; Student's t-test) 6 h after FSL-1 application compared to DPBS vehicle. By 24 h, induced cytokines had returned to DPBS vehicle levels. Each collection time point is presented as mean ± SEM of five replicates from two independent experiments.

Previous work with other TLR agonists or cytokine knockout mice showed that secretion of specific cytokines correlated with resistance to genital HSV-2 challenge in mice [15-17,19]. To compare established cytokine profiles to the one produced by FSL-1, vaginal lavage samples from mice treated with FSL-1 or equal volumes of the DPBS vehicle were collected at 0, 6, 24 and 48 h post-application. Individual lavages then were analyzed to quantify specific cytokines that were significantly increased by FSL-1 treatment compared to DPBS vehicle. The FSL-1 profile, evident by 6 h, included significant (p < 0.05; Student's t-test) induction of IL-1α, IL-1β, IL-2, IL-6, IL-12(p40), IL-12(p70), G-CSF, GM-CSF, IFNα, IFNβ, IFNγ, MIP-1α, MIP-1β and TNF-α (Table 3). Cytokine induction profiles produced by a single 2 or 6 μg dose were indistinguishable (data not shown). Lavage samples collected at later time points showed that the induced cytokines had returned to levels similar to DPBS vehicle controls within 24 h (Figure 1). Overall, the observed cytokine profile was consistent with the profiles elaborated by other TLR agonists that significantly reduced genital HSV-2 infection in mice [15-17,19].

**Table 3:** Vaginal application of FSL-1 in mice induced a specific cytokine profile.

	6 h Post FSL-1 application (pg/lavage ± SEM)	
	
Cytokine^a^	FSL-1	DPBS vehicle
IL-1α	1083.2 ± 143.5^b^	527.0 ± 65.7
IL-1β	1157.0 ± 320.2^c^	169.2 ± 46.3
IL-2	34.8 ± 5.2^d^	BDL(3.9)^e^
IL-6	418.5 ± 94.1^b^	35.4 ± 8.5
IL-12(p40)	104.2 ± 10.5^d^	44.9 ± 3.8
IL-12(p70)	64.0 ± 19.1^c^	17.1 ± 3.1
G-CSF	5157.0 ± 1095.0^c^	2208.0 ± 433.1
GM-CSF	198.6 ± 44.6^b^	22.6 ± 8.7
IFNα	30.3 ± 4.4^d^	0.7 ± 0.6
IFNβ	445.3 ± 93.2^b^	13.8 ± 3.5
IFNγ	76.8 ± 18.8^c^	BDL(43.1)
MIP-1α	2770.7 ± 484.0^b^	440.0 ± 127.0
MIP-1β	4140.0 ± 570.5^c^	2232.0 ± 218.5
TNF-α	1109.6 ± 212.7^b^	232.6 ± 71.6

^a ^The indicated cytokines were found to be induced by FSL-1 application by BioPlex or ELISA as indicated in the methods. Data are presented as mean ± SEM of five replicates from two independent experiments.

^b ^p < 0.01 compared to DPBS vehicle (Student's t-test).

^c ^p < 0.05 compared to DPBS vehicle (Student's t-test).

^d ^p < 0.001 compared to DPBS vehicle (Student's t-test).

^e ^BDL; Below Detection Limit (for statistical comparisons with these cytokines the value for the lowest standard of each cytokine was utilized).

### Multiple FSL-1 doses applied vaginally did not induce chronic inflammation or organ enlargement

**Figure 2:**
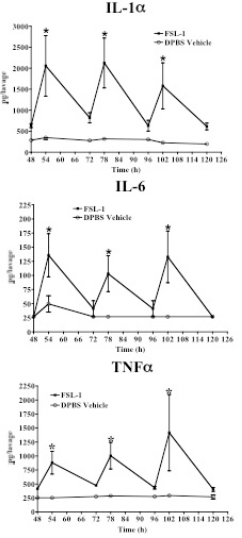
**FSL-1 delivered once daily for 5 consecutive days did not induce chronic inflammation**. As a marker of an inflammatory state, IL-1α, IL-6 and TNF-α were kinetically quantified in murine lavages of repetitive daily dosed animals (n = 5/treatment). Each cytokine was induced significantly (*, p < 0.05; Student's t-test) 6 h after FSL-1 (6 μg; closed square) application compared to DPBS vehicle (60 μL; open circle). Notably, by 24 h after each FSL-1 dose, induced cytokines had returned to DPBS vehicle levels and were not significantly (p > 0.05; Student's t-test) increased 24 h after the final FSL-1 application. Each collection time point is presented as mean ± SEM of five replicates from two independent experiments.

**Figure 3:**
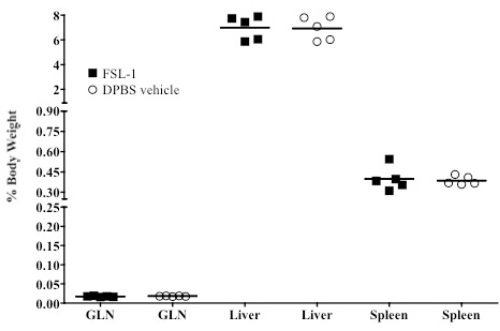
**Single or multiple FSL-1 dosing did not induce organ enlargement**. As an additional measure of peripheral immunotoxicity, immune cell migration was evaluated by gross observation of associated lymphoid tissues following FSL-1 application. GLN, liver and spleen weights for organs collected 2 or 5d after delivery of FSL-1 (6 μg; closed square) or DPBS vehicle (60 μL; open circle) in a single or 5 multiple once daily doses. By organ, the mean % of total body weight for each group is demarcated by the horizontal line with each point representing one mouse (n = 5/group). Weights of organs collected 2d after the last of 5 consecutive daily FSL-1 doses were not significantly (p > 0.05; Student's t-test) different than organs collected from mice treated with an equivalent volume of the DPBS vehicle. Similar statistically (p > 0.05; Student's t-test) indistinguishable results were observed for organs collected 2 or 5d after a single FSL-1 dose (data not shown). Groups were compared for statistical significance by one-way ANOVA.

Effective anti-herpetic compounds should only transiently induce cytokines even after multiple dosing cycles [17,30]. To determine if FSL-1 caused chronic cytokine elaboration, vaginal lavages were collected at 6 and 24 h following daily 6 μg FSL-1 applications delivered over five consecutive days. The 6 μg dose was selected because it was the highest tested dose that elicited resistance to genital HSV-2 infection. Cytokine production was similar to the single dose results (Figure 1) following the first dose and showed significant (p < 0.05; Student's t-test) induction of the indicated cytokines 6 h after FSL-1 application (Table 3) with a return to DPBS vehicle levels by 24 h (Figure 2). The cycle of induction and return to DPBS vehicle levels was observed after each daily dose of FSL-1 (Figure 2). Importantly, no additive effect of repeated FSL-1 dosing was observed; cytokine levels 24 h after the last dose (120 h) were indistinguishable from DPBS vehicle treated mice (Figure 2). As an additional preliminary evaluation of potential chronic inflammation, the weights of genital lymph nodes (GLN), liver and spleen (n = 5 mice/group) were determined at indicated time points after delivery of a single or five consecutive daily 6 μg doses of FSL-1. GLN, liver and spleen weights from treated mice were statistically (p > 0.05; Student's t-test) indistinguishable from DPBS vehicle treated mice (Figure 3). Together, the data indicate that repetitive 6 μg doses of FSL-1 did not elicit chronic cytokine elaboration or cause organ enlargement unlike the outcomes observed following CpG application in mice [16].

### FSL-1 reduced HSV-2 replication in human vaginal EC

**Figure 4:**
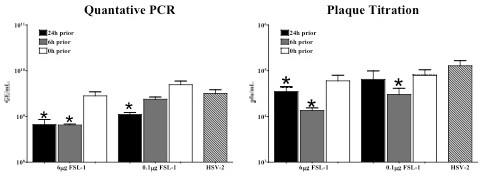
**FSL-1 significantly reduced HSV-2 replication in vitro**. Triplicate human vaginal EC cultures were treated with FSL-1 (6 μg or 0.1 μg) at 24 h (black bar), 6 h (grey bar) or just prior (white bar) to HSV-2 inoculation (10^4^pfu/well). A separate set of cultures were treated with the DPBS vehicle, infected and processed in parallel (hatched bar). Following 24 h of viral infection, each well was collected (100 μL) and 50 μL of the sample was used for standard plaque titration assays. DNA was extracted from the remaining sample (50 μL) and subjected to quantitative PCR to measure the amount of viral replication. FSL-1 (6 μg) delivered 24 or 6 h prior to infection significantly (*, p < 0.05; student's t-test) reduced viral replication compared to the DPBS vehicle for both PCR and plaque titration analyses. For the 0.1 μg FSL-1 dose, significant (*, p < 0.05; student's t-test) reduction in GE was observed 24 h prior to HSV-2 inoculation while significant (*, p < 0.05; student's t-test) reduction in pfu was observed 6 h prior to viral challenge. Addition of FSL-1 at either dose just prior to infection did not impact HSV-2 replication. Results are presented as the mean ± SEM from two separate experiments using three vaginal EC lines (V11I, V12I, V19I) in each experiment.

The ability of FSL-1 to elicit anti-herpetic activity in human vaginal EC was quantified to confirm that the observed reduction in genital HSV-2 infection was not specific to the mouse model and to provide data on the role of genital EC in the resistance process. FSL-1 at the higher 6 μg dose or at a previously tested 0.1 μg in vitro dose [13] was added to triplicate human vaginal EC cultures 24, 6 h or just prior to HSV-2 inoculation (10^4^pfu/well). Virus control wells received an equal DPBS vehicle volume at the indicated times. PCR was used to quantify reduction in HSV-2 genome equivalents (GE) for the glycoprotein B gene and showed that FSL-1 (6 μg) produced significant (p < 0.05; Student's t-test) reductions when delivered at 24 or 6 h prior to HSV-2 inoculation (Figure 4). A similar significant (p < 0.05; Student's t-test) reduction in viral replication was measured using the plaque titration assay. The 0.1 μg FSL-1 dose produced reduced HSV-2 replication at 24 or 6 h prior to viral challenge (Figure 4). Three separate EC donors were tested in parallel and produced comparable outcomes. Similar to results obtained in the mouse model, FSL-1 added just prior to HSV-2 inoculation did not significantly (p > 0.05; Student's t-test) reduce viral infection (Figure 4) confirming that FSL-1 was not inhibiting HSV-2 directly.

## Discussion

For the first time, these murine studies illustrated a synthetic bacterial-derived TLR2/6 agonist, FSL-1, significantly increased resistance to genital HSV-2 infection. The observed increase in resistance has potential implications for HSV-2 transmission prevention by increasing the threshold of infection above the amount of virus shed during a sexual encounter [31,32]. FSL-1 (2 μg) application at 24 h prior to viral inoculation engendered the greatest resistance to experimental HSV-2 challenge of the tested doses (25-fold increase over the DPBS vehicle ID_50_) (Table 2). Based on recent epidemiological studies, even an anti-herpetic strategy that did not completely protect against but induced significant resistance to infection could substantially reduce HSV-2 transmission [33]. To confirm that the observed activity of FSL-1 was not specific to the murine model, evaluations also were preformed using a model of the human vaginal mucosa.

FSL-1 also elicited significant anti-herpetic activity in human vaginal EC. The EC of the vaginal mucosa are the initial targets of HSV-2 [1,34] and actively participate in the innate immune response against infection [11,13]. Additionally, human and murine vaginal EC both express high levels of TLR2 and 6 [13,23]. A significant or trended reduction in HSV-2 GE and pfu was observed following application of FSL-1 24 or 6 h prior to viral challenge (Figure 4). Interestingly, no anti-herpetic activity was observed for FSL-1 applied just prior to HSV-2 infection. The observed temporal dependant activity is consistent with the mechanism of action for previously evaluated anti-herpetic TLR agonists [14,15,17,19].

The FSL-1-induced increase in resistance to genital HSV-2 infection in mice is similar to the activity observed for other anti-herpetic TLR agonists. Application of PIC, IMI or CpG prior to viral challenge elicited significant resistance to genital HSV-2 infection while PGN, LPS or FLAG showed no activity [14,15,17,19]. The HSV-2 genome is composed of double-stranded DNA that is recognized by TLR9 [11,24,35]. Also, viral replication potentially produces double- or single-stranded RNA intermediates that are recognized by TLR3 or TLR7/8, respectively [11,24,36,37]. There are no published reports showing HSV-2 recognition through TLR5 and recent data indicated that PGN recognition occurs through NOD1/2 or TLR2 [11,38,39]. Interpretation of the results would suggest that induction of anti-herpetic activity is specific to viral-associated agonists, but recent work by Ashkar et al. showed that FimH, a TLR4 agonist derived from *E. coli*, provided significant resistant to genital HSV-2 infection while LPS from *E. coli *was ineffective [20]. Additionally, we showed that FSL-1, a bacterial-derived diacyl lipopeptide [21,22], significantly increased resistance to HSV-2 infection in mouse and human models. The varying anti-herpetic activity observed following activation of different TLR pathways indicates that effective agonists likely induce a specific innate immune response profile that produces an HSV-2 resistant environment.

FSL-1 elaborated a cytokine response profile consistent with other anti-herpetic TLR agonists. Vaginal application of CpG in mice resulted in elaboration of IL-12, IL-18, IFNγ and RANTES [15,16,19]. The cytokine profile elicited by PIC or IMI vaginal treatment was similar to CpG and included secretion of IL-1α, IL-1β, IL-6, IL-12, IFNβ, IFNγ, MIP-1α and RANTES [15,17,19]. Interestingly, vaginal delivery of PGN or FLAG in mice did not result in production of IFNα or IFNβ [15]. Based on the observed cytokine profile elicited by FSL-1 6 h post-application (Table 3) and other in vitro or in vivo experiments [15-17,19,40-45], a preliminary anti-herpetic cytokine profile would include IL-2, IL-12(p40), IL-12(p70), IFNβ, IFNγ, MIP-1α, MIP-1β and RANTES.

The identified cytokines are important for providing resistance to genital HSV-2 infection through direct anti-herpetic activity or through stimulation and activation of leukocytes. Gill et al. and others showed that IFNβ production correlated with protection of mice against experimental genital HSV-2 infection [15,19]. Type I interferons (IFNα, IFNβ) act by directly inhibiting HSV-2 replication and skew the immune system towards a T_H_1-baised response [42,44,46]. A T_H_1-associated immune response is important for controlling early replication of HSV-2 and for effective clearance of the infection [42,44,47]. Elaboration of IL-2 and IL-12 also aids the innate immune response against HSV-2 through stimulation and activation of leukocytes and induction of type II interferon (IFNγ) [16,40,45]. Resistance to HSV-2 is enhanced by the direct anti-herpetic activity of MIP-1α, MIP-1β and RANTES [43]. The chemokines also are important for the recruitment of leukocytes that aid in the regulation of the anti-herpetic immune response [41,43].

Importantly, the TLR agonist-induced innate immune response against HSV-2 infection must provide adequate resistance without producing chronic inflammation that can potentially increase acquisition of STI. Kinetic vaginal cytokine elaboration and organ weights following FSL-1 application were evaluated and showed no chronic or systemic outcomes. Chronic inflammation could result in disruption of the vaginal mucosal barrier leading to increased susceptibility to STI [30] so careful analyses of prolonged inflammation are necessary. Our initial studies indicated that even repeated dosing with FSL-1 did not create a chronically inflamed vaginal tract (Figure 3). Also, the treatment regimen resulted in no enlargement of the GLN, spleen or liver (Figure 4). PIC application in mice produced similar results but specific pro-inflammatory cytokines remained elevated until 48 h post-application [17]. Further, Ashkar et al. showed that delivery of CpG resulted in significant enlargement of the spleen in mice due to a large influx of immune cells [14]. The observed systemic immunotoxicity following CpG application highlights the need for a TLR agonist that not only provides resistance to genital HSV-2 infection but does not negatively impact the vaginal mucosa or associated lymphoid tissues [30]. Comparison of the preliminary FSL-1 safety profile to other TLR agonists confirmed that the mouse model is useful for assessing the preliminary safety profile of immunomodulatory compounds prior to evaluation in expensive and lengthy clinical trials.

In summary, we showed that the TLR2/6 agonist FSL-1 is capable of significantly increasing resistance to HSV-2 infection in mice through induction of a specific cytokine response profile. The identified anti-herpetic profile is of critical importance for the future design of TLR agonist interventions and will aid in improving the efficacy of promising adjuvants for HSV-2 vaccines [48,49]. Additionally, the vaginal EC-elicited innate immune response following FSL-1 application provides intriguing rationale for investigation into the impact of secondary reproductive tract infections by genital mycoplasmas or other potential coinfecting pathogens on HSV-2 transmission. Indeed, HSV-2 is associated with increased susceptibility to other important bacterial and viral STI including HIV-1 [1,5-7]. Continued investigation into the innate response to specific TLR agonists like FSL-1 will provide a greater understanding of how pathogens are recognized in the polymicrobial environment of the vagina and how these responses can be exploited to develop novel strategies for STI prevention.

## Conclusion

• The bacteria-derived FSL-1 TLR2/6 agonist applied vaginally to mice increased resistance to HSV-2 challenge.

• The observed FSL-1 anti-herpetic resistance may indicate that pre-existing colonization by *Mycoplasma spp *or other bacteria, including commensals, that produce diacyl lipopeptides could alter HSV-2 susceptibility.

• Application of FSL-1 to human vaginal epithelial cells reduced HSV-2 replication.

• Based on the cytokines produced by FSL-1, PIC and CpG TLR agonists it appears that IL-2, IL-12(p40), IL-12(p70), IFNβ, IFNγ, MIP-1α, MIP-1β and RANTES contribute to resistance to HSV-2.

## Competing interests

The authors declare that they have no competing interests.

## Authors' contributions

WAR was responsible for planning and conducting experiments, data analysis and manuscript preparation. CLM assisted in planning and conducting experiments involving evaluation of HSV-2 anti-herpetic activity in mice. RBP designed the research program, provided project guidance, reviewed data analyses and interpretations and assisted in manuscript preparation.
